# The diverse functions of FAT1 in cancer progression: good, bad, or ugly?

**DOI:** 10.1186/s13046-022-02461-8

**Published:** 2022-08-15

**Authors:** Zhuo Georgia Chen, Nabil F. Saba, Yong Teng

**Affiliations:** Department of Hematology and Medical Oncology, Winship Cancer Institute, Emory University School of Medicine, 201 Dowman Dr, Atlanta, GA 30322 USA

**Keywords:** FAT1, Cancer progression, Gene mutations, Signaling regulatory network, Targeted treatment

## Abstract

FAT atypical cadherin 1 (FAT1) is among the most frequently mutated genes in many types of cancer. Its highest mutation rate is found in head and neck squamous cell carcinoma (HNSCC), in which FAT1 is the second most frequently mutated gene. Thus, FAT1 has great potential to serve as a target or prognostic biomarker in cancer treatment. *FAT1* encodes a member of the cadherin-like protein family. Under normal physiological conditions, FAT1 serves as a molecular “brake” on mitochondrial respiration and acts as a receptor for a signaling pathway regulating cell–cell contact interaction and planar cell polarity. In many cancers, loss of FAT1 function promotes epithelial-mesenchymal transition (EMT) and the formation of cancer initiation/stem-like cells. However, in some types of cancer, overexpression of FAT1 leads to EMT. The roles of FAT1 in cancer progression, which seems to be cancer-type specific, have not been clarified. To further study the function of FAT1 in cancers, this review summarizes recent relevant literature regarding this protein. In addition to phenotypic alterations due to FAT1 mutations, several signaling pathways and tumor immune systems known or proposed to be regulated by this protein are presented. The potential impact of detecting or targeting FAT1 mutations on cancer treatment is also prospectively discussed.

## Background

The challenges in effectively treating head and neck squamous cell carcinoma (HNSCC) are attributed to its extreme heterogeneity as far as anatomic locations and genetic aberrations [1–4]. These genetic alterations, especially gene mutations, which accumulate during the growth of cancer, create difficulties in understanding the biology of the disease and lead to ineffective and non-targeted approaches which can only go so far in altering the prognosis of patients. The Cancer Genome Atlas (TCGA) and other omics-based studies have provided the most comprehensive characterization to date of the genomic and proteomic landscape in many types of cancers. *FAT atypical cadherin 1* (*FAT1*) is among the group of genes that is most frequently mutated in many cancers. For example, in TCGA dataset, the *FAT1* mutation rate is around 10–18% in both lung adenocarcinoma (LUAD) and lung squamous cell carcinomas (LUSCC), esophageal cell carcinoma (ESCC), and cervical SCC [5]. The highest mutation rate was found to be ~ 23% in HNSCC, ranking as the second most mutated gene after *TP53* in this disease and suggesting its important role in the biology of HNSCC [3, 4]. In HPV-negative (HPV-) HNSCC, the *FAT1* mutation rate is as high as 28% with many truncation and nonsense mutations [6, 7], suggesting that wild-type *FAT1* serves as a tumor suppressor gene in this disease [2]. These observations were also supported by some publications. Martin et al.reported that the *FAT1* gene alteration rate is as high as 29.8% in HNSCC, which is the highest among solid tumors [5]. *FAT1* mutation was reported to be more common in HPV-negative than in HPV-positive HNSCC (28% vs. 2.8%) [6]. In a study from Taiwan, 29% of HNSCC had *FAT1* mutation [8]. Mann et al.examined 16 HNSCC cell lines and reported a *FAT1* mutation rate of 43% [9]. Table 1 summaries *FAT1* gene mutations in HNSCC cell lines from the CCLE Cancer Cell Line Encyclopedia (https//site.broadinstitute.org/ccle/datasets) [9–12].

**Table 1:** FAT1 gene status in various HNSCC cell lines

Cell line primary name	Variant Classification	Variant Type	Anato Class	Anatomy	Node Status	Gender	HPV
BHY	Nonsense_Mutation	SNP	OC	Alveolus	Negative	M	Negative
BICR 18	Silent	SNP	LX	Larynx	Positive	M	N/A
	Frame_Shift_Del	DEL	-	-	-	-	-
	Frame_Shift_Ins	INS	-	-	-	-	-
BICR 31	Nonsense_Mutation	SNP	OC	Tongue	Positive	M	N/A
BICR 56	Nonsense_Mutation	SNP	OC	Tongue	Positive	F	N/A
BICR78	Splice_Site	SNP	OC	Oral alveolus	N/A	M	N/A
CAL-33	Nonsense_Mutation	SNP	OC	Tongue	Negative	M	Negative
FaDu	Frame_Shift_Del	DEL	OC	Hypopharynx	Positive	M	Negative
H357	Frame_Shift_Del	DEL	OC	Tongue	N/A	M	N/A
	Nonsense_Mutation	SNP	-	-	-	-	-
H376	Missense_Mutation	SNP	OC	Floor of mouth	N/A	F	N/A
HO1N1	Nonsense_Mutation	SNP	OC	Baccul Muc	N/A	N/A	N/A
HO1U1	Nonsense_Mutation	SNP	OC	Mouth Floor	N/A	N/A	N/A
HSC-2	Missense_Mutation	SNP	OC	Floor of mouth	Negative	M	N/A
HSC-3	Frame_Shift_Del	DEL	OC	Tongue	Positive	M	Negative
JHU022	Missense_Mutation	SNP	LX	Larynx	Positive	M	Negative
MDA686TU^a^	Missense_Mutation	SNP	OP	Base of Tongue	Positive	M	Negative
OSC19	Frame_Shift_Del	DEL	OC	Tongue	Positive	M	N/A
PE/CA-PJ41	Missense_Mutation	SNP	OC	N/A	N/A	F	N/A
PE/CA-PJ49	Nonsense_Mutation	SNP	OC	Tongue		M	N/A
	Missense_Mutation	SNP	-	-	-	-	-
SCC-15	Missense_Mutation	SNP	OC	Tongue	N/A	M	Negative
SNU-46	Frame_Shift_Del	DEL	LX	Larynx	N/A	M	Negative
SNU-1041	Missense_Mutation	SNP	OC	Pharynx	N/A	N/A	N/A
	Nonsense_Mutation	SNP	-	-	-	-	-
SqCCY1^a^	Frame-Shift-Ins	INS	OC	N/A	N/A	M	Negative
UMSCC1^a^	Missense_Mutation	SNP	OC	Floor of mouth	Negative	M	Negative
UMSCC9	Missense_Mutation	SNP	OC	Tongue	Negative	F	Negative
UMSCC11A	Missense_Mutation	SNP	OC	Epiglottis	Positive	M	Negative
UMSCC25	Frame-Shift-Del	DEL	LN	Lymph Node	Positive	M	Negative
UMSCC28	Nonsense_Mutation	SNP	-	True cord	Negative	F	Negative
UMSCC41	Missense_Mutation	SNP	-	Arytenoid	Positive	M	Negative
UMSCC74A	Missense_Mutation	SNP	OP	Base of Tongue	Negative	M	Negative
UMSCC74B	Missense_Mutation	SNP	Recurrence	Intraoral	Negative	M	Negative
UMSCC76	Missense_Mutation	SNP	LN	Lymph Node	Positive	M	Negative
UMSCC81B	Missense_Mutation	SNP	-	True cord	Negative	M	Negative
UMSCC104	Missense_Mutation	SNP	OC	Floor of mouth	Positive	M	Positive
UPCISCC116	Frame_Shift_Del	DEL	OC	ALV Ridge	N/A	M	Nagative
YD-10B	Frame_Shift_Del	DEL	OC	Tongue	N/A	M	N/A

Most of the information can be found in CCLE (https//site.broadinstitute.org/ccle/datasets)

^a^ FAT1 status were determined by our group

The function of FAT1 in both normal and cancer tissues has been studied since FAT1 was discovered in Drosophila [13, 14]. However, FAT1 seems to be playing different roles in different tissues or cancer types based the finding that its expression level is upregulated in acute leukemia, hepatocellular carcinoma (HCC), glioblastoma (GMB), and gastric cancer, but downregulated in HNSCC, ESCC, breast cancer, and cervical cancer [15], though one recent study reported upregulation of FAT1 in oral cancer [16]. Particularly, the effect of *FAT1* mutation on development of malignant phenotypes has not been extensively investigated, and little is known about its clinical implications. The objective of this review is to summarize the potential functions of FAT1 and its mutations in cancer progression to facilitate the development of treatments for patients harboring this specific mutated or deleted protein. HNSCC has been a focus in recent literature since this disease carries the highest *FAT1* mutation rate among solid tumors [5].

## Main text

### Biology of normal FAT1 as an adhesion molecule

**Fig. 1:**
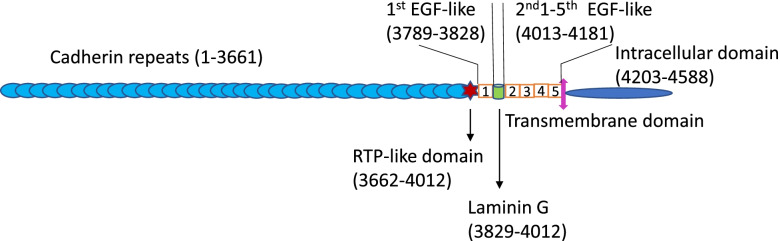
Human FAT1 protein structure

The human *FAT1* gene was cloned from the human T-cell acute lymphoblastic leukemia (T-ALL) cell line in 1995 and is located on chromosome 4q34-35 and consists of 27 exons [17]. FAT1 is a cadherin-like protein family member, as a large type 1 transmembrane protein that encodes 4588 amino acid residues. It has 34 cadherin repeats, a laminin G domain, and five epidermal growth factor (EGF)-like repeats in the extracellular region, followed by a transmembrane region and a C-terminal cytoplasmic tail containing a PDZ-binding motif [18, 19] (Fig. 1). Early studies identified FAT1 as an ortholog of the Drosophila *fat* gene family. Under normal physiological conditions, FAT1 serves as a molecular “brake” on mitochondrial respiration [20], which regulates proliferation and migration of vascular smooth muscle cells in case of injury [21, 22]. It also acts as a receptor for a signaling pathway regulating cell–cell contact interaction and planar cell polarity [23, 24]. FAT1 has been found to be involved in the development of certain vertebrates and some hereditary diseases, such as eye abnormalities [15]. Loss of *fat* leads to cell cycle dysregulation and hyperproliferation in *Drosophila* larval imaginal discs [25]. Studies on regulation of *FAT1* gene expression under normal physiological condition are limited, but two studies have identified transcriptional activator binding elements responding to NFκB and E2F1 in *FAT1* gene promoter region [26, 27]. In addition to these functions in regulating normal cell activities, FAT1 plays roles in blocking or facilitating carcinogenesis and cancer progression depending on the cancer type.

### FAT1 as a potential tumor suppressor

Since FAT1 is downregulated in many types of cancer and plays a role in controlling cell proliferation and migration, it has been considered a tumor suppressor. Strong evidence supporting this came from a transgenic mouse model. Pastushenko et al.performed conditional deletion of *Fat1* in the skin epidermis using a *Fat1*-constitutive knockout (*Fat1*-cKO) mouse model [28]. After *Fat1*-cKO mice were born, DMBA/TPA was applied to the skin. The number of benign and malignant tumors per mouse was noted to increase in *Fat1*-cKO mice, suggesting that *Fat1* acts as a tumor-suppressor gene in DMBA/TPA-induced skin SCCs. Immunohistochemistry analyses revealed that in *Fat1*-cKO mice, the polarity of the basal cells as well as the adherens and tight junctions were rapidly lost. In this study, the authors also confirmed their observations using combined deletion of *Fat1* with *p53* and *kRASG12D* expression in the lung epithelia by intratracheal installation of Cre-expressing adenovirus. It was found that *Fat1* deletion considerably increased the number of tumors per lung tissue [28].

Tumor initiation is suggested to occur from cancer initiating cells that are also called stem-like cells. Pastushenko et al.identified stemness features in the *Fat1*-cKO cells, which was supported by an increased numbers of spheroids in *Fat1*-knockout as compared with *FAT1* wild-type cells [28]. In another study, Li et al.reported that overexpression of wild-type FAT1 decreased stem-like cell markers and suppressed formation of spheroids in non-small cell lung cancer (NSCLC) cells [29]. They demonstrated that FAT1 might reduce the tumor-initiating ability in NSCLCs by promoting Yes-associated protein 1 (YAP1) nuclear-cytoplasmic translocation [29].

### FAT1 as a potential cancer type-specific metastatic suppressor or promoter

In addition to the suppression of tumor initiation, FAT1 may also suppress metastasis. Multiple lines of evidence have suggested that inactivation of FAT1 results in epithelial-mesenchymal transition (EMT) through a variety of signaling pathways [28, 30–33]. In an ESCC study, overexpression of FAT1 led to inhibition of cell proliferation and colony formation, as well as cell migration and invasion. FAT1 knockdown led to a dramatic decrease in E-cadherin expression along with increased N-cadherin, vimentin, and snail mediated by MAPK/ERK signaling, while overexpression of FAT1 resulted in the opposite trends [32]. Jiang et al.reported that S100A14 suppressed proliferation and EMT in prostate cancer [30]. It turned out that S100 calcium binding protein A14 (S100A14) promoted the expression of FAT1 and activated the Hippo complex activity, which, therefore, suppressed prostate cancer progression. Depletion of FAT1 reversed the suppression of cell proliferation and EMT resulting from S100A14 overexpression in prostate cancer. These observations were confirmed in a PC3 prostate cancer xenograft mouse model [30]. More convincingly, in a *Fat1*-cKO mouse model, the proportion of spontaneous lymph node and lung metastases and the number of metastases per mouse were increased as compared with wild-type control. Also, intravenous injection of EPCAM^+^ *Fat1*-cKO tumor cells gave rise to a higher number of lung metastases as compared to tumor cells with wild-type FAT1, which clearly illustrated that deletion of FAT1 promotes metastasis in vivo [28].

In contrast, in some types of cancers, FAT1 may facilitate metastasis. GBM is characterized by the presence of hypoxia, stemness and local invasiveness. It was found that markers of EMT (Snail/LOX/Vimentin/N-cad), stemness (SOX2/OCT4/Nestin/REST) and hypoxia (HIF-1a/VEGF/PGK1/CA9) were upregulated in 39% of GBM tumors with significant positive correlation with the expression of FAT1, consistent with the data from TCGA. FAT1 knockdown in U87MG/A172 maintained in severe hypoxia primary glioma cultures led to significant reduction of EMT/stemness markers as compared to the controls, suggesting FAT1 is a regulator of EMT/stemness in hypoxic GBM [33]. A recent mechanistic study has identified an interaction between glypican-3 (GPC3), which is a surface heparan sulfate proteoglycan and FAT1 in HCC cells [31]. The GPC3 binding region on FAT1 was mapped to the C-terminal region (Q14517, residues 3662–4181), which covered a putative receptor tyrosine phosphatase (RTP)-like domain, a laminin G-like domain, and five EGF-like domains. Expression of both GPC3 and FAT1 in HCC were upregulated under hypoxia conditions, and thus were able to upregulate the expression of snail, vimentin, and downregulate E-cadherin, promoting HCC cell migration. However, these studies did not have supportive data from animal models of metastasis.

### Function of circular FAT1

Several recent studies have identified circular FAT1 (circFAT1) in cancer cells. circFAT1 is a non-coding RNA with a cyclic structure which was initially reported in osteosarcoma in 2018 [34]. It is formed by the back-splicing of exon 2 of the FAT1 gene and head-to-tail binding. Like cell surface FAT1, circFAT1 has a dual-function, either inhibiting or promoting tumor progression in a cancer specific manner through sponging miRNAs and repressing their downstream pathways [35–40]. In ESCC, downregulation of circFAT1expression by siRNA promoted ESCC cell migration and invasive ability, but not proliferation. Consequently, the expression of miR-548 g was upregulated, which promoted ESCC cell migration and invasion [37].

In contrast, in HNSCC, Jia et al.screened 4573 circRNA in tumors and identified circFAT1 as one of 6 highly expressed circRNAs associated with shorter overall survival (OS) [35]. They further revealed that one of the mechanisms for circFAT1 to promote HNSCC progression was through binding with STAT3 and subsequently inhibiting this signaling pathway. Knockdown of circFAT1 by siRNA in HNSCC cell lines reduced tumorsphere formation in vitro and tumor growth in vivo. Interestingly, they also found that knockdown of circFAT1 significantly enhanced the efficacy of PD1 immunotherapy by enhancing CD8^+^ infiltration into tumor tissues. Additionally, circFAT1 expression is upregulated in HCC tissues and cells and positively correlated with TNM stage and tumor size [40]. Depletion of circFAT1 by siRNA repressed the proliferation and invasion of HCC cells in vitro and tumorigenesis in vivo. circFAT1 sponges miR-30a-5p to downregulate the expression of REEP3 and inhibits HCC proliferation and invasion, blocking hepatocarcinogenesis.

### Potential contributions of FAT1 to immune regulation

Currently, there are limited publications regarding FAT1’s regulation of the cancer immune system. A notable publication is from Feng et al.who recently reported a high mutation rate of FAT1/2/3/4 (57.3%, 603/1052) in NSCLC patients [41]. They found that LUAD patients with FAT1 mutations showed significantly high infiltration of activated dendritic cells, while those with FAT2/3/4 mutations had high infiltration of CD8^+^T-cells, M1 macrophages, activated memory CD4^+^ T-cells, and helper follicular T-cells. They also found that FAT1/2/3 mutations were associated with longer progression-free survival in an immune checkpoint inhibitor (ICI)-treated NSCLC cohort. FAT1/4 mutations were related to better OS in pan-cancer patients treated with ICIs. Another study on NSCLC was consistent with their observation and further suggested that high FAT1 mutation rate is associated with high tumor mutation burden (TMB), which could be used to predict patient response to ICIs [42]. These observations were supported by a recent study on 631 melanoma and 109 NSCLC samples [43], which showed that patients with melanoma and NSCLC harboring FAT1 mutations had favorable outcomes from ICI therapy. Genomic and immunologic analysis showed that a high TMB, increased infiltration of immune-response cells, decreased infiltration of immune-suppressive cells, interferon and cell cycle-related pathways were enriched in patients with FAT1 mutations.

Investigations from fields unrelated to cancer have yielded some interesting clues regarding FAT1’s effects on the immune regulatory system. Studies from wild-type *FAT1* gene transgenic mice showed downregulated gene expression of TNF-α, IL-6, NF-kB, and CCL2 as compared with non-transgenic wild-type mice, supporting that overexpression of FAT1 may downregulate these inflammatory cytokines/chemokines [44]. In contrast, mutated FAT1 may upregulate growth factors and proinflammatory cytokines, such as TGFB1 [45], IL-6 [46, 47], and FGF2 [48], consistent with our recent findings [49]. This mechanism may be mediated by YAP1, which is activated by FAT1 inactivation [5]. Activated YAP1 has been found to upregulate CCL2 in endothelial cells [50, 51].

However, a preliminary TIMER2.0 analysis of TCGA database revealed an inverse correlation of FAT1 expression with infiltration of tumor-inhibiting immune cells (*e.g.,* monocytes and T cells) and a positive correlation with myeloid-derived suppressor cells (MDSCs) in GBM, HCC, cervical and pancreatic cancers, which is also positively correlated with TGFB1/2 expression and eventually results in immune suppression [52]. FAT1 knockdown in GBM primary cultures and cell lines led to a reduction in TGFB1/2 expression/secretion. These findings are consistent with the contribution of FAT1 to the progression in these cancers.

### Abnormal signaling transduction of mutated FAT1

**Fig. 2:**
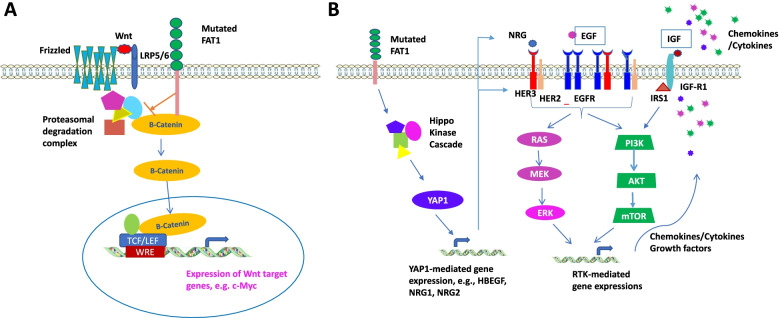
Major signaling pathways affected by mutated FAT1. **A** Wnt/β-catenin signaling pathway: FAT1 can bind to β-catenin. Mutated FAT1 releases β-catenin from proteasomal degradation complex, which enhances the nuclear translocation and transcriptional activity of β-catenin. **B** Hippo/YAP1 activation and receptor tyrosine kinase (RTK) signaling pathways: Mutated FAT1 releases YAP1 from Hippo complex and activates it as a transcription factor. Mutated FAT1 also enhances HER3 activation and IRS1 expression, which may contribute to activation of multiple RTK signaling pathways. A potential link between YAP1 and ERBB signaling may be due to an autocrine loop through their ligand EGF and NRGs

Due to its numerous biological activities in cell growth and cell–cell interaction, FAT1 is involved in the regulation of many signaling pathways. Mutation of FAT1 results in dysregulation of these signaling transductions, which potentially contributes to carcinogenesis and cancer progression (Fig. 2).

#### Wnt/β-catenin signaling pathways

Previous studies of the functional impact of *FAT1* mutation were focused on the activation of Wnt signaling pathways. Activation of Wnt/β-catenin signaling pathway includes three steps: Wnt transducing signal at the membrane, stabilizing β-catenin in the cytoplasm, and activating Wnt/β-catenin target genes in the nucleus. FAT1 protein may affect Wnt signaling via enhancing β-catenin activity. Morris et al.reported that endogenous FAT1 bound to β-catenin in human cells [11]. In experiments using glioma cells and immortalized human brain astrocytes, they found that knockdown of FAT1 resulted in a decrease in plasma membrane β-catenin staining, and a significant increase in nuclear β-catenin translocation. Therefore, inactivated FAT1 expression could alter gene expression mediated by Wnt/β-catenin pathway components.

#### Hippo/YAP1 activation

The Hippo/YAP1 pathway is one of the key oncogenic regulatory pathways in multiple cancers including HNSCC [6]. FAT1 is one of the cell surface modulators involved in the regulation of YAP1 activation. A recent study by Martin et al.endorsed this observation [5]. They found that wild-type FAT1 assembled a multimeric Hippo signaling complex (signalome) which is required for activation of core Hippo kinases by TAOKs and consequent YAP1 phosphorylation. Phosphorylated YAP1 was inactive. When FAT1 was mutated, YAP1 was not restrained by phosphorylation, it then acted as an oncogenic driver in HNSCC and contributed to aggressiveness, suggesting that targeting YAP1 may serve as an attractive precision therapeutic option for cancers harboring genomic alterations in FAT1 tumor suppressor genes. In a study of ECC, Lu et al.revealed that FAT1 and PTPN14 regulated malignant progression and chemotherapy resistance through the Hippo/YAP1 signaling pathway [53]. In addition, there is reported evidence that YAP1 signaling, which was activated by *FAT1* mutation [5], can activate EGFR family members though upregulation of their ligands in ovarian cancer [54], which suggests a potential linkage between YAP1 and EGFR signaling. Recently, Chen et al.reported that the YAP1/TAZ/TEAD transcriptional complex recruits BRD4 to promote an active chromatin state and regulate multiple oncogenic transcriptional programs in HNSCC [55]. Therefore, FAT1 mutated HNSCC exhibits selective and higher sensitivity to BRD4 inhibition.

#### EGFR family/MAPK/ERK signaling pathways

Whether the EGFR/MAPK signaling pathway is regulated by FAT1 through YAP1 has not been reported. Previous studies initially observed that in pituitary spindle cell tumor, FAT1 mutations were associated with increased ERK activity, highlighting an association between FAT1 and the ERK signaling pathway [56]. A later study in ECC also reported that knockout of FAT1 significantly increased the levels of p-ERK1/2, while overexpression of FAT1 decreased p-ERK1/2 levels [32].

In contrast, we recently reported that knocking out *FAT1* in HNSCC cell lines significantly reduced p-ERK, which may be a consequence of EGFR inactivation [49]. These observations were consistent with *FAT1* transgenic mouse studies [28]. Through a biostatistics/bioinformatics study using HNSCC TCGA proteomic database, we also observed increased cell surface proteins, such as HER3_pY1289, VEGFR2, and PDL1, plus IGFR signaling mediator IRS1 and cell cycle modulator CMYC in more than 90 HNSCC patient samples with *FAT1* mutation. Specifically, our data showed that in both total and HPV(-) patients, HER3_pY1289 was upregulated in *FAT1* mutated HNSCC. HER3 is one of the EGFR family members, with a high affinity of binding to the growth factor neuregulin, and HER3_pY1289 is the activated form that transduces signaling after partnering with other EGFR family members [57, 58]. In addition, IRS1 [59], a key regulator of IGF-1R, is also upregulated in *FAT1* mutated HNSCC. Cross-talk between HER3 and IGF-R1 [60] signals could synergistically activate ERK/MAPK, PI3K/AKT, and RAS/RAF pathways and promote cell proliferation/survival, protein synthesis, and cell cycle through CMYC [61]. In contrast, similar downstream functions and biological categories are affected by different receptors and signaling molecules in tumors containing wild type *FAT1*. HER2_pY1248 [57] and RET_pY905 [62] are surface receptors upregulated through activated signaling molecules, including SRC_pY527 [63], SHC_pY317 [64], MTOR_pS2448 [65], and transcription factors CJUN_pS73 [66, 67], which could synergistically activate ERK/MAPK, PI3K/AKT, and RAS/RAF pathways, and promote cell proliferation/survival and protein synthesis. P16INK4A [68] and CYCLINE2 [69] regulate the cell cycle, while PAI1 [70] promotes cellular angiogenesis, migration, and invasion abilities. ASNS [71] and SCD1 [72] modulate protein and lipid metabolisms, affecting cell cycle, proliferation, and apoptosis.

#### Interaction between FAT1 and actin cytoskeletal dynamics

Actin dynamic is important in governing cell migration and cell–cell interaction. It has been reported that FAT1 cytoplasmic domain recruits components of actin such as Ena/VASP and Homer1/3 proteins that regulate the actin polymerization complex [13, 73, 74]. FAT1 knockdown decreased recruitment of endogenous VASP to the leading edge of the cell and resulted in weakening of lamellipodial dynamics, stopping of polarization, and controlling cell migration. Ena/VASP was found to be involved in migration of breast cancer cells [75]. Actin-mediated cellular cytoskeletal dynamics has been linked to cancer cell progression and metastasis [76, 77]. Most likely, inactivated FAT1 may regulate cancer progression and metastasis partially through the interaction with Ena/VASP and Homer-mediated cellular cytoskeletal dynamics [78].

#### Other cancer related proteins affected by FAT1

In our TCGA study, IPA analysis revealed the dominant upstream regulators, including TP53 and CMYC, as potentially activated transcription factors and cell surface molecules. In addition, Madan et al. revealed that under hypoxic conditions, depletion of endogenous FAT1 could reduce the expression of HIF1a and its downstream target genes such as CA9, GLUT1, VEGFA, MCT4, HK2, BNIP3 and REDD1. Consequently, a significant reduction in invasiveness was observed in GBM cells [79]. This observation is consistent with the study by Srivastava et al.who suggested FAT1 promoted EMT under hypoxia [33]. It is worth mentioning that Hayes et al.reported synergistic effects of FAT1 and CASP8 inactivation on migration and colony formation of oral cancer cell lines [80] since both are frequently mutated in this type of cancer [81]. The mechanism behind this observation remains to be determined.

### Perspective: clinical significance of FAT1 as a prognostic marker or treatment target

As a frequently mutated protein in cancer, several studies have examined FAT1 as a cancer prognostic biomarker. In an earlier study, FAT1 was found to be overexpressed in paired diagnosis-relapse samples of precursor B-cell acute lymphoblastic leukemia. High FAT1 mRNA expression was correlated with shorter relapse-free and overall survival, and it was independent of other traditional prognostic markers, such as white blood cell count, sex and age in this disease [82]. In the last 10 years, several publications have reported a correlation between FAT1 mutation or expression with prognosis in different type of cancers, such as breast cancer [83], NSCLC [41], gastric cancer [84], and T-cell lymphoma [85]. Using HNSCC as an example, the Taiwanese study on HNSCC by Lin et al.showed significant correlations of *FAT1* mutations with lymph node status and worse disease-free survival (DFS) [8]. Kim *et a.* examined 566 HNSCC patients who were classified into FAT1-associated low risk (FAT1-LR; *n* = 195) and FAT1-associated high risk (FAT1-HR; *n* = 371) subgroups. The five-year overall survival and recurrence-free survival rates were significantly lower in the FAT1-HR subgroup than in the FAT1-LR subgroup (*P* = 0.01 and 0.003, respectively). These results were validated using four independent cohorts [86]. It is worth mentioning that most oropharyngeal squamous cell carcinomas (OPSCCs) are HPV ( +), while FAT1 mutation occurs dominantly in HPV(-) SCC. Harbison et al.reported that metachronous recurrent OPSCCs share similar genomic features with HPV-unrelated HNSCC including FAT1 mutations [87], implicating a potential role of FAT1 mutation in recurrent HNSCC regardless of HPV status. Our recent study using TCGA proteomic database also demonstrated that HNSCC patients with FAT1 mutations had a shorter progression-free survival than those with wild-type FAT1 [49]. These studies support that FAT1 represents a promising prognostic biomarker. It is expected that a standard must be established for each cancer type based on additional studies with validations in multiple cohorts.

FAT1 expression or mutation has also been linked to cancer treatment sensitivity. Lepikhova et al.screened 45 HNSCC cell lines for sensitivity to EGFR, MEK, and mTOR inhibitors [88]. They found that cell lines harboring a stop-gain mutation in FAT1 showed a tendency for higher sensitivity to the mTOR inhibitor temsirolimus as compared with other cell lines. Pastushenko et al.examined the sensitivity of wild-type and isogenic *FAT1*-knockout human cancer cell lines to targeted inhibitors and found *FAT1*-knockout cells were significantly more resistant to the EGFR/HER2 inhibitor afatinib and MEK inhibitor trametinib as compared to *FAT1* wild-type cells in vitro [28]. In contrast, *FAT1*-knockout tumor cells were significantly more sensitive to the SRC inhibitor dasatinib and SRC/Bcr-Abl inhibitor saracatinib and the CAMK2 inhibitor KN93 as compared to *FAT1* wild-type cells. Administration of afatinib and dasatinib to mice transplanted with *FAT1* wild-type and knockout human SCC cell lines showed that *FAT1* wild-type tumor cells were more sensitive to afatinib and *FAT1*-knockout tumor cells were more sensitive to dasatinib, consistent with the differences in drug sensitivity observed in vitro. Li et al.performed genomic analysis of 348 estrogen receptor-positive breast cancer patients treated with CDK4/6 inhibitor and found that loss of function mutation of FAT1 led to resistance to the CDK4/6 inhibitor through the Hippo/YAP1 signaling pathway [89]. Interestingly, in a recently completed phase II clinical trial using a combination of HER3 inhibitor CDX-3379 and EGFR inhibitor cetuximab in recurrent/metastatic, HPV-negative, cetuximab-resistant HNSCC, the investigators analyzed tissues from 27 patients including one of two responders. They reported that the overall response rate was 1/10 (complete response; 10%; 95% CI 0.30–44.5) in the FAT1-mutated versus 0/17 (0%; 95% CI: 0–19.5) in the FAT1-wildtype cohorts, suggesting that FAT1 mutation may play a role in resistance to EGFR-targeting therapy through activation of HER3. Upon further follow up these correlations did not however hold upon completion of the trial [90]. FAT1 mutation may be an indicator for outcome in these specific combination strategies. A recent study by Zhai et al. showed that FAT1 downregulation in ESCC enhanced stemness and reduced patients’ sensitivity to cisplatin [91]. They found that knockdown of FAT1 could induce multi-drug resistant protein ABCC3 due to FAT1 mediated nuclear translocation of β-catenin. It seems that the effect of FAT1 on drug sensitivity is related to aggressive behaviors, such as stemness and EMT status of cancer cells. The contributions of FAT1 mutations to tumor and the tumor microenvironment interaction, particularly to the immune regulatory system, have not been clarified. These interactions may substantially affect tumor cell response to cancer therapy in the human body, which deserves further studies.

FAT1 is expected to define a new subclass of HNSCC. However, no agents are currently available to target FAT1 directly due to lack of fully understanding of FAT1 mutation sites and their functional alterations as compared with the wild-type protein. Our proteomic analysis has provided basic information that surface receptors and signaling molecules, such as HER3 phosphorylation, are associated with FAT1 mutation. On the bright side, some of these molecules are druggable with commercially available agents, such as HER3-DXd, a novel HER3 directed antibody drug conjugate, and Vertepofin, a YAP1 inhibitor interrupting the interaction between YAP1 and TEAD [92], which can be considered to suppress the oncogenic pathways mediated by FAT1. Kang et al.found that FAT1 was overexpressed in gastric cancer [84]. They demonstrated that verteporfin could suppress FAT1 expression, leading to decreased migration and invasion of gastric cancer cells. Recently, Gutkind’s group summarized genomic alterations in the Hippo pathway and persistent YAP/TAZ activation in HNSCC. Since the mutation frequency of FAT1 is high in this disease, blocking this pathway may provide novel multimodal precision therapies for HNSCC. We also suggest multiple strategies which can indirectly target this Hippo/YAP1 pathway [93], including use of EGFR inhibitor cetuximab, PI3K inhibitor alpelisib, MEK inhibitor trametinib, FAK inhibitor defactinib, tankyrase inhibitors, SRC inhibitor dasatinib, YAP1 inhibitor verteporfin, and smTEAD inhibitor. Most recently, Chen et al.performed comprehensive proteomic and drug-screening studies across pan-cancer models and confirmed that FAT1 mutated HNSCC had selective and higher sensitivity to BRD4 inhibition by JQ1 which has been also used to block YAP1 signaling [55]. Another approach is to disrupt the FAT1-associated protein complex by pre-designed stapled peptides. We have gained experience in developing this type of therapeutic peptide in the past [94–96] and are now working on identifying novel points of susceptibility for peptide intervention. No doubt, there is a long way to go. Still, it is imperative to further the knowledge regarding the impact of FAT1 mutations on cancer development at the molecular and therapeutic levels, as well as discover novel FAT1-targeted therapeutic strategies for personalized medicine.

## Conclusion

The *FAT1* mutation rate in HNSCC is the highest among major solid tumors, making its investigation of primary interest in this disease [49]. Although a high FAT1 mutation rate has been identified in many types of cancer, its functions and clinical significance remain to be further elucidated. Particularly, the current literature brings some conflict and contradiction regarding the function of this protein. Whether FAT1 serves a tumor suppressor or promoter seems to be cancer type-specific. Furthermore, since *FAT1* is a gene with no identified mutation hot spots as seen for *TP53*, mutation-specific functional alterations of this protein remain to be discovered, which will be essential for targeting this protein in precision cancer therapy.

## Data Availability

Not applicable.

## Abbreviations

circFAT1: Circular FAT1
DFS: Disease-free survival
EGF: Epidermal growth factor
EMT: Epithelial-mesenchymal transition
ESCC: Esophageal cell carcinoma
*Fat1*-cKO: *Fat1*-Constitutive knockout
GMB: Glioblastoma
GPC3: Glypican-3
HCC: Hepatocellular carcinoma
HNSCC: Head and neck squamous cell carcinoma
ICI: Immune checkpoint inhibitor
LUAD: Lung adenocarcinoma
LUSCC: Lung squamous cell carcinomas
NSCLC: Non-small cell lung cancer
MDSCs: Myeloid-derived suppressor cells
OPSCCs: Oropharyngeal squamous cell carcinomas
OS: Overall survival
RTK: Receptor tyrosine kinase
RTP: Receptor tyrosine phosphatase
S100A14: S100 calcium binding protein A14
T-ALL: T-cell acute lymphoblastic leukemia
TMB: Tumor mutation burden
YAP1: Yes-associated protein 1
